# Hepatic GIST metastases: an illustrative case series

**DOI:** 10.1259/bjrcr.20210166

**Published:** 2022-01-10

**Authors:** Cathryn Hui, Reuben Sum

**Affiliations:** 1 Department of Diagnostic Imaging, Monash Medical Centre, Melbourne, Australia; 2 Monash University, Melbourne, Australia

## Abstract

Gastrointestinal stromal tumours (GISTs) are uncommon mesenchymal tumours affecting the gastrointestinal tract. The liver is one of the most common sites for metastatic disease from GISTs and may exhibit a variety of CT and MR imaging appearances. These imaging features can vary prior to and following treatment with tyrosine kinase inhibitors. We report on the spectrum of imaging appearances of hepatic GIST metastases on multiphase contrast CT imaging and hepatocyte-specific contrast enhanced MR. To our knowledge, there are no published series specifically focusing on the appearances of liver metastases from GISTs.

An awareness of the protean appearances and pitfalls on CT and MRI of hepatic GIST metastases, prior to and at different times along the treatment pathway, will assist in early diagnosis of liver metastases, accurate assessment of tumour response and detection of recurrent metastatic disease.

## Background

Gastrointestinal stromal tumours (GISTs) are the most frequent mesenchymal neoplasm occurring in the gastrointestinal tract. Although GISTS are uncommon, the incidence seems to be increasing which may be related to an elevated awareness and improved histopathological detection of these types of neoplasms.^1^

GISTs arise from the interstitial cells of Cajal, mesenchymal stem cells located in the myenteric plexus, which serve as a pacemaker controlling gastrointestinal peristalsis.^2^ The most common sites for GISTs are the stomach (up to 60% of cases) and small bowel (up to 30% of cases). GISTs can also arise from extraintestinal sites, including the mesentery, omentum and retroperitoneum.^3^

Pathologically, the diagnosis of GISTs is based on morphological features as well as immunohistochemical findings, with CD117 (KIT) and/or DOG1 positivity. In approximately 5% of GISTs, immunohistochemical analyses for CD117/DOG1 are negative and genetic testing may confirm the diagnosis. Mutation analysis is useful to predict response to molecular-targeted therapy and it also provides prognostic information.^4^

Most GISTs occur in older adults with a median age of 60–65 years and slight male predominance.^4^ These conventional GISTs are driven by gain-of-function mutations in KIT or RTK PDFGRA (platelet derived-growth factor receptor α mutations in the receptor tyrosine kinase) leading to increased kinase signaling.^5^ KIT mutations are found in 80% of GISTs and involve the related receptor PDGFRA in approximately 10% of cases.^5^

10–15% of GISTs without KIT/PDFGRA mutations are known as ‘RTK-wild-type GISTs’ (WT-GISTs). These encompass a variety of tumours that include paediatric GISTs, those associated with syndromes (such as those in Carney triad and Neurofibromatosis Type 1), and those associated with BRAF mutations.^6^ Succinate dehydrogenase (SDH) enzyme complex deficiency is seen in approximately 40% of these GISTs that lack RTK mutations. They result from genetic inactivation of one of the four SDH subunit genes (SDHA, SDHB, SDHC, SDHD, collectively referred to as SDHX).^5^ These GISTs may be seen sporadically in young adults or in multitumour syndromes. They almost exclusively occur in the stomach and have a greater tendency for lymphovascular invasion, lymph node involvement, and liver metastases, although slow progression of the metastases occurs.^5,7^

The treatment paradigm for GISTs has evolved rapidly over the last 15 years due to imatinib mesylate, a tyrosine kinase inhibitor.^8^ Imatinib is now the first-line therapy for GISTs and has a high specificity for the cKIT proto-oncogene. It is associated with an 80% response rate.^9^ The median survival time, prior to use of tyrosine kinase inhibitors, was less than 16 months.^8^ This has increased substantially to 5 years upon introduction of tyrosine kinase inhibitors, although acquired resistance to imatinib from secondary gene mutations has been reported in almost 50% of patients after 18 months.^9–11^ The mutational status of these oncoproteins predicts clinical response to imatinib.^11^ For example, patients with KIT exon 11 point mutations respond well to imatinib and have a more favourable prognosis.^12^ The RTK-wild type-GISTs respond poorly to imatinib treatment.^5^

Multiphase CT abdomen and pelvis is advised for staging and follow-up. CT chest is performed in the initial assessment. Fludeoxyglucose-positron emission tomography/CT may be useful to detect early tumour response to molecular targeted therapy or when surgical resection of metastases is considered.^4^ Surveillance imaging protocols vary among different institutions and are influenced by risk assessment based on the mitotic count, tumour size and site. As an example, for high-risk patients with resected primary GISTs, ESMO guidelines suggest follow-up with abdominal CT or MRI every 3–6 months for 3 years, during adjuvant therapy, then 3-monthly for 2 years on cessation of adjuvant therapy, followed by 6-monthly for 5 years from cessation of adjuvant therapy and annually for an additional 5 years.^4^

At diagnosis, up to 50% of patients will present with metastatic disease.^13^ The most common locations for metastatic GISTs are the liver and peritoneum.^1^ The majority of patients undergoing complete resection of their primary GIST will develop tumour recurrence at a median time of 18–24 months. At recurrence, approximately two-thirds of these patients exhibit liver metastases and half will develop peritoneal disease.^14^ We present a series of radiological images that depict a range of appearances of GIST liver metastases at different time points along the treatment pathway.

## Pre-treatment CT appearance

GIST liver metastases may be hyper- or hypovascular. The frequency of each of these enhancement patterns is not well understood as this has not adequately been evaluated in the literature. There are few studies involving small numbers of cases. In a retrospective study by Patnaik et al, the CT imaging features of 12 cases of GIST liver metastases were evaluated: five were heterogeneously enhancing, two cases were hypodense, four lesions exhibited predominantly cystic attenuation with thin peripheral enhancement. One lesion contained haemorrhage. It was not evident whether multiphase CT imaging was performed, nor in which phases these observations were made.^2^ In our series, the site and size of the primary lesion was not predictive of the enhancement characteristics of the liver metastases.

Hypervascular liver metastases are best detected in the arterial phase.^15^ Choi et al suggest imaging GIST liver metastases with multiphasic CT imaging, which includes an arterial phase, as the lesions may become isoattenuating in the portal venous phase and therefore, difficult to visualise without arterial phase imaging.^16^ Arterial phase imaging is also useful to monitor changes in tumour vascularity before and after treatment. Varying opinions regarding the use of multiphasic or portal venous phase imaging alone are discussed in the current literature. The German GIST Imaging Working Group suggests initial triphasic CT which consists of a non-enhanced phase, arterial phase, and a portal venous phase of the liver with the portal venous phase covering the complete abdomen and pelvis. The group advises a non-contrast and portal venous CT study during treatment and a triphasic CT at the end of therapy.^3^

## Hypervascular GIST liver metastases

### Case 1

**Figure 1.**
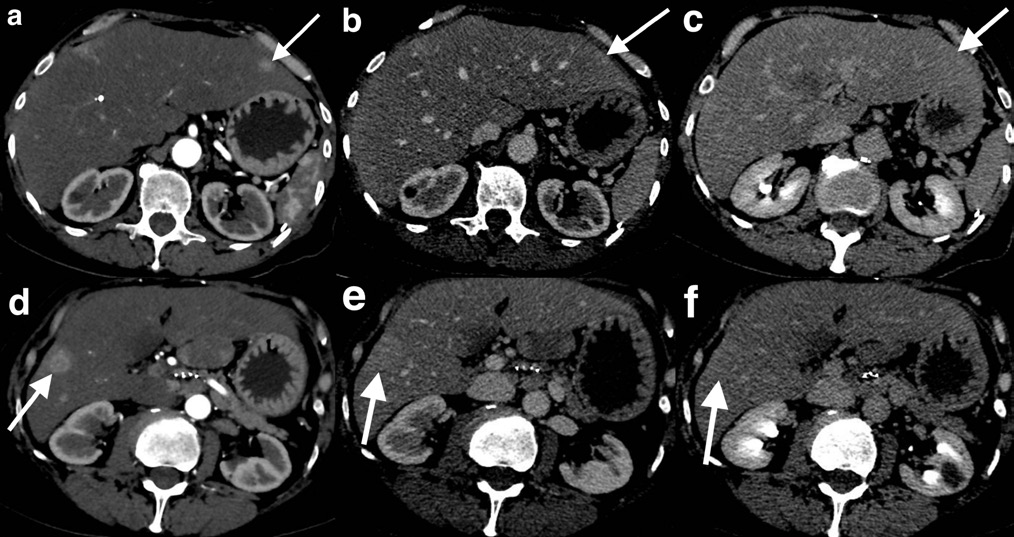
Hypervascular GIST liver metastases. Multiphase CT demonstrates a segment two lesion which diffusely enhances in the arterial phase (**a**). It is difficult to see in the portal venous phase and appears subtly hyperattenuating (**b**). It is isoatttenuating in the delayed phase (**c**). A second lesion at the junction of segments 5 and 6 exhibits rim enhancement in the arterial phase (**d**). In the portal venous phase, it shows mildly diffuse hyperattenuation with a wedge-shaped perfusion abnormality (**e**). In the delayed phase, the lesion is isoattenuating with a subtly enhancing rim (**f**). GIST, GIST, gastrointestinal stromal tumour/.

A 65-year-old male presented 2 years post-resection of jejunal GIST with the development of new liver lesions on a surveillance CT. The resection of the primary lesion was complicated by mesenteric artery thrombosis, requiring near-total small bowel resection. Tyrosine kinase inhibitor therapy had not been administered due to compromised absorption of oral medication. A multiphase CT demonstrated a segment two lesion with diffuse arterial phase enhancement (Figure 1). The lesions were suspected to represent hypervascular liver metastases secondary to the GIST, which was subsequently confirmed on biopsy.

## Hypovascular GIST liver metastases

### Case 2

**Figure 2.**
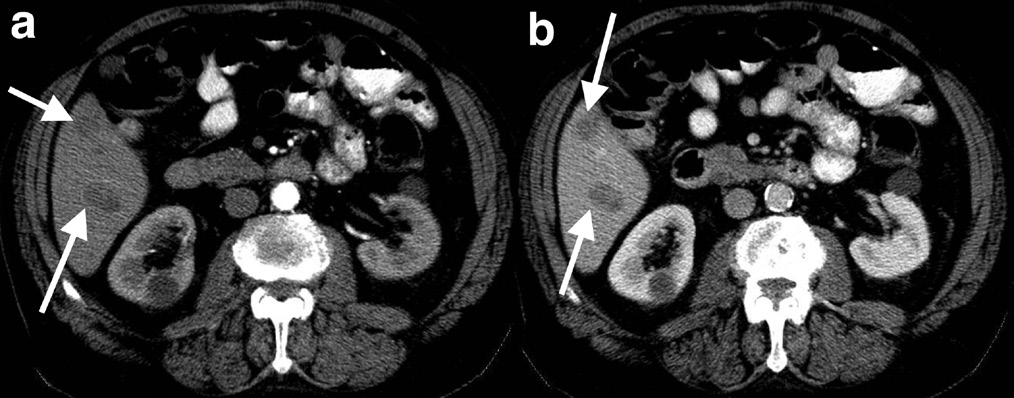
Hypovascular GIST liver metastases. Multiphase CT images demonstrates segment 5 GIST liver metastases are hypoattenuating (arrows) in the arterial phase (**a**) and portal venous phase (**b**). GIST, gastrointestinal stromal tumour.

A 78-year-old male who presented with abdominal pain was found to have a gastric GIST and liver metastases. Multiphase CT demonstrates segment seven liver metastases which are hypoattenuating in both the arterial and portal venous phases (Figure 2).

## MRI appearances

**Figure 3.**
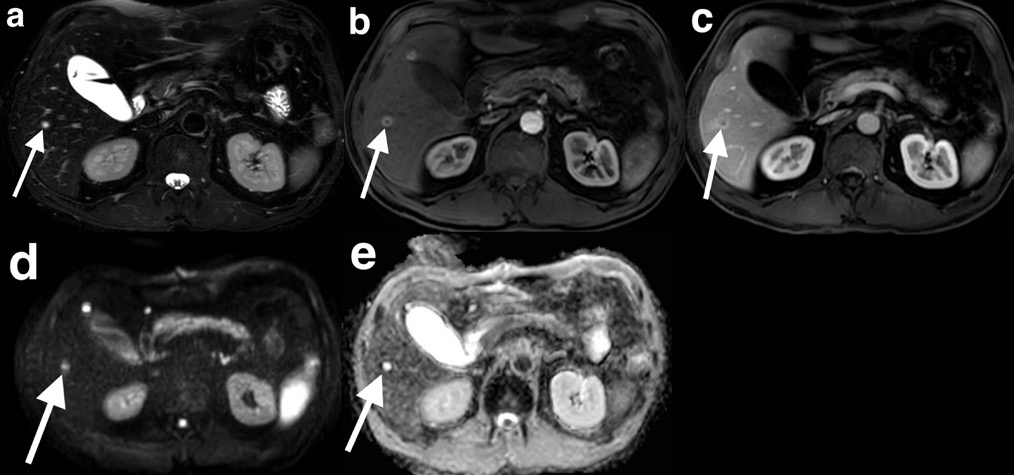
Gadoxetic acid-enhanced MRI showing diffusion weighted imaging appearances. A *T*_2_ weighted image demonstrates a marked T2 hyperintense lesion in segment 6 (white arrow) (**a**). In the arterial phase, there is a thick rim of vivid enhancement. A second, similarly enhancing lesion in segment five is located adjacent to the gallbladder (red arrow) (**b**). The enhancement persists in the portal venous phase (**c**). There is no restricted diffusion, with high signal intensity demonstrated on both the diffusion-weighted sequence (**d**) and ADC map (**e**). ADC, apparent diffusion coefficient.

MRI of the liver is recommended in cases when liver resection is considered or if there is a contraindication to CT contrast.^3^ Liver MRI with hepatocyte-specific contrast has the highest sensitivity for detection of liver metastases.^17^ There is a paucity of information within the current literature regarding the MRI appearances of GIST liver metastases. In our series, four patients (11 lesions) with newly diagnosed GIST liver metastases who underwent MRI with gadoxetic acid, all lesions demonstrated hypointensity on *T*_1_ weighted imaging, hyperintensity on *T*_2_ weighted imaging and hypointensity in the delayed hepatobiliary phase (Figure 3).

Diffusion-weighted sequences are sensitive to microscopic motion of water molecules and in turn, reflect tissue cellullarity. A lower apparent diffusion coefficient (ADC) value may correlate with the higher cellularity of malignant tumours, potentially predicting malignancy risk.^18^ Most liver metastases demonstrate restricted diffusion (reduced ADC values) and treatment response is associated with an increase in ADC values.^19^ In our series, however, the diffusion characteristics were variable. In two of four patients with newly diagnosed GIST liver metastases who underwent MRI, the liver lesions did not demonstrate restricted diffusion prior to commencing tyrosine kinase therapy (Figure 3). We hypothesise that this may be related to cystic change within GISTs, which may occasionally be the predominant pathological finding, related to liquefactive necrosis, haemorrhage and myxoid changes.^20^ Hepatic metastases comprising central necrosis are known to demonstrate high ADC values.^21^ The observation of increased ADC in tumours post-treatment may be less helpful in such cases when the ADC is high prior to treatment.

### Case 3

**Figure 4.**
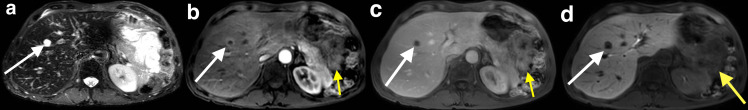
MRI appearances of GIST liver metastases. Gadoxetic acid-enhanced MRI reveals a segment 8 GIST metastasis which is hyperintense on *T*_2_ weighted imaging (**a**). In the arterial phase, there is a halo of enhancement (**b**). The metastasis is hypointense in the portal venous (**c**) and 20 min hepatobiliary phase MRI (**d**). The gastric GIST can also be seen (yellow arrows). GIST, gastrointestinal stromal tumour.

A 59-year-old male who presented with abdominal pain and weight loss was found to have a gastric mass at gastroscopy. Biopsy confirmed a KIT mutated GIST. CT and MRI revealed a segment 8 lesion which was avid on fludeoxyglucose CT positron emission tomography (not shown). On the gadoxetic acid enhanced MRI, the lesion is T2 hyperintense. It is markedly T2 hyperintense and demonstrates a rim of arterial phase enhancement, hypointensity in the portal venous phase and hypointensity in the hepatobiliary phase (Figure 4). The diffusion-weighted sequences show facilitated rather than restricted diffusion.

### Case 4

A 49-year-old male presented with abdominal pain and was found to have a jejunal mass and liver lesions on CT. The jejunal mass was resected and found to be a KIT mutated GIST. Gadoxetic acid-enhanced MRI was performed. Multiple lesions exhibited hyperintensity on *T*_2_ weighted imaging and rim enhancement in the arterial phase. The lesions enhanced in the portal venous phase. Despite the study being performed prior to commencing imatinib, the lesions did not demonstrate restricted diffusion (Figure 3).

## Post-treatment changes

Traditionally for any liver metastases, RECIST (Response Evaluation Criteria In Solid Tumours) criteria are used to assess treatment response by monitoring tumour burden based on size criteria. The Choi criteria were developed with the use of Imatinib in the treatment of advanced metastatic GIST as there was no linear correlation between treatment efficacy and size of the tumour. Choi criteria define a partial response to treatment by either a 10% reduction in size or a 15% reduction in density of the lesion during the portal venous phase of contrast.^22^ Tumour shrinkage is usually minimal during the early stages after treatment, whereas alterations in internal characteristics are more indicative of treatment response.^23^

**Figure 5.**
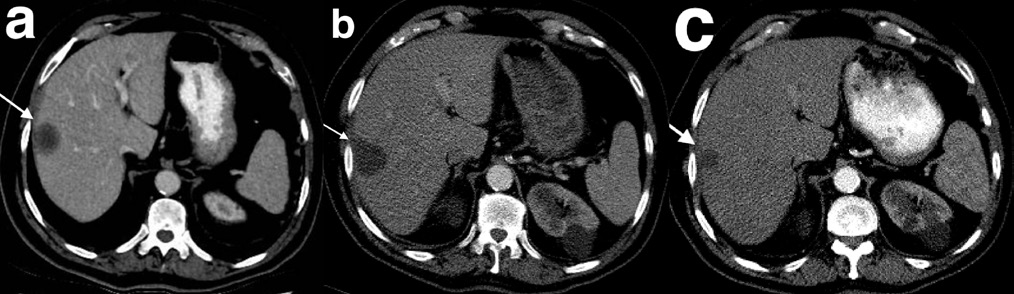
Post-treatment appearances of GIST liver metastases. Portal venous CT performed prior to treatment demonstrates a lesion in segment 5 of the liver which is hypoattenuating with a central area of more marked hypoattenuation (**a**). Following tyrosine kinase inhibitor therapy, there is a reduction in density from 36 to 9 Hounsfield units and a mild increase in size (**b**). A further portal venous CT 18 months after the initial CT shows that the lesion remains cystic in attenuation and has decreased in size (**c**). GIST, gastrointestinal stromal tumour.

**Figure 6.**
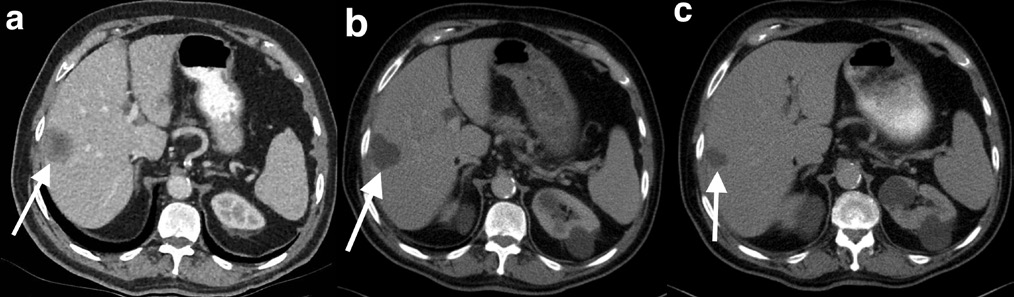
Post-treatment appearances of GIST liver metastases. Portal venous CT demonstrates a hypoattenuating segment 5/8 of the liver (**a**). 6 months after commencement of tyrosine kinase inhibitors, the lesion becomes more hypoattenuating but increases in size (**b**). Despite no change in treatment regimen, CT performed 12 months after the initial scan demonstrates reduction in size of this lesion (**c**). GIST, gastrointestinal stromal tumour.

Treatment with imatinib results in a decrease in tumour attenuation and reduction in heterogeneity.^24^ GIST liver metastases may undergo cystic degeneration after treatment which may not necessarily be accompanied by a reduction in size (Figure 5). GISTs may even enlarge during treatment - this pseudoprogression is attributed to haemorrhage, necrosis, or myxoid degeneration^23^ (Figure 6). Accordingly, volumetric assessment of tumour will have similar limitations to one- and two-dimensional quantification of GIST liver metastases, as other indicators of tumour viability such as tumour density are not accounted for. Volumetric analysis provides an objective three-dimensional size assessment of lesions. It has been evaluated in assessment of post-treatment changes in breast and colorectal liver metastases but has not been utilised in the evaluation of GIST hepatic metastases. There is potential utility of volumetric assessment when viability-based criteria are incorporated, such as assessment of volume of enhancing tumour volume, which has been evaluated in the setting of hepatocellular carcinoma.^25^

Dual energy CT (DECT) is useful in improving conspicuity and detection rate of liver metastases. DECT utilises two different energies allowing material decomposition on the basis of energy-dependent attenuation profiles of specific materials. Post-processing yields a variety of images, including low-energy virtual monochroic images and material-specific iodine images, which increase conspicuity of iodine, assisting in detection and characterisation of tumours. Use of virtual monochromatic images of a specific energy level improves lesion-to-background contrast. Material-specific iodine images enables differentiation of hypoattenuating tumour from cysts and assists in detection of isoattenuating tumours. Quantitative iodine mapping could potentially serve as a surrogate biomarker for monitoring treatment effects. This technique may potentially quantify tumour viability post-treatment; however, this is yet to be validated and research in this area is ongoing.^26^

### Case 5

An 83-year-old male presented 10 years after resection of a pelvic GIST. The patient commenced imatinib post-operatively but suffered a severe skin rash and was not able to tolerate the medication. He ceased taking imatinib approximately 9 years prior to presentation and was lost to follow-up. This patient presented with pelvic pain and was found to have a recurrent pelvic mass and new liver lesions. Portal venous CT performed prior to treatment demonstrates a lesion in segment 5 of the liver which is hypoattenuating with a central area of more marked hypoattenuation. He was commenced on sunitinib, a second-line tyrosine kinase inhibitor. A subsequent CT at 6 months shows a slight increase in size of the lesion, however, the density of the lesion has reduced. Despite the increase in size of the lesion, the reduction in density of the lesion indicates treatment response. A further portal venous CT performed 18 months later demonstrates a persistently low attenuation lesion which has also reduced in size (Figure 5).

### Case 6

An 82-year-old male with resected gastric GIST developed a new lesion on surveillance CT. The lesion exhibits hypoattenuation in the portal venous phase. He commenced imitanib therapy and the lesion becomes more hypoattenuating on a CT 7 months later, indicating treatment response. This was despite an increase in size of the lesion.

A further CT performed 12 months after the initial scan demonstrates reduction in size of this lesion (Figure 6).

## Recurrent GIST liver metastases

**Figure 7.**
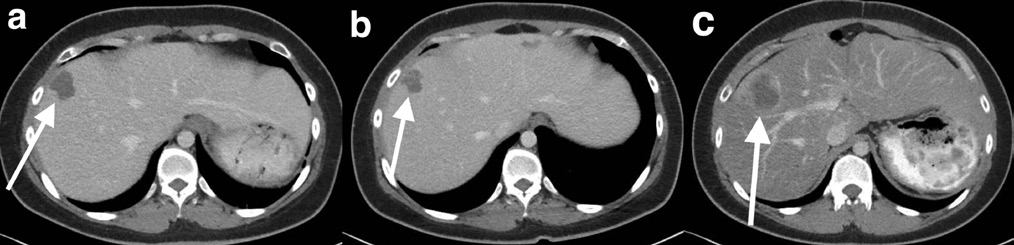
Recurrent liver metastases. Portal venous CT image demonstrates a treated segment 8 liver metastasis with expected cystic attenuation (a). CT performed 3 months later shows a similar sized lesion with new areas of internal enhancement anteriorly and centrally (b). A third CT performed 6 months later demonstrates marked increased density and size increase in size in keeping with recurrent metastatic disease related to second-line tyrosine kinase inhibitor resistance (c).

**Figure 8.**
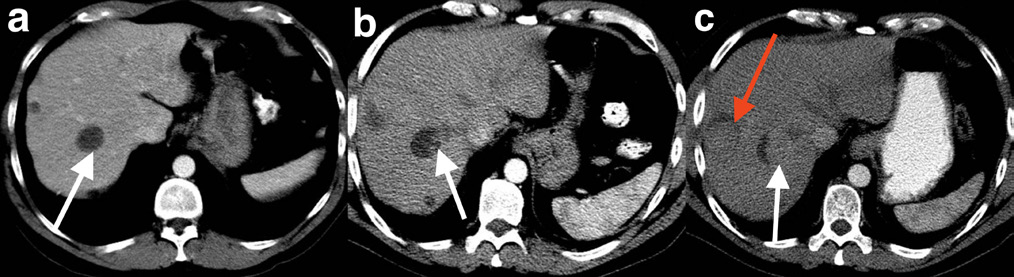
Recurrent liver metastases. Portal venous CT shows treated cystic attenuation of GIST liver metastases - the largest is located in segment 7 (**a**). CT performed 3 months later demonstrates subtle nodularity within the largest lesion (**b**). This was not recognised. A study 3 months later shows increase in size of the nodule within the cystic metastasis (white arrow) as well as recurrence of a smaller peripheral segment 7 lesion only partially imaged (red arrow) (**c**). GIST, gastrointestinal stromal tumour

Recurrence of hepatic metastases from GISTs can be suspected when there is an increase in size or density of treated lesions or development of a new lesion.^27^ An increase in size is important, however recurrence may occur in a treated hypoattenuating metastasis without a change in tumour size.^23^ Recurrence can also manifest as a new area of enhancement (Figure 7) or the development of intratumoral nodules - referred to as a ‘nodule within mass’ pattern of progression (Figure 8).^28^ Increase in tumour density due to haemorrhage, especially with sunitinib therapy, may mimic tumour progression.^29^

### Case 7

A 30-year-old female presented 3 years after a resection of a gastric GIST requiring partial gastrectomy and splenectomy. She was subsequently commenced on imatinib. Routine surveillance portal venous CT image demonstrates a treated segment 8 liver metastasis with expected cystic attenuation. A further CT performed 3 months later shows a similar sized lesion, however, it contains areas of internal enhancement. These findings were not appreciated at the time, therefore there was no change in treatment regimen. A third CT performed 6 months later demonstrates marked increase in size and increased attenuation of the lesion with further areas of enhancement in keeping with recurrent metastatic disease related to imatinib resistance (Figure 7). Following this, she commenced sunitinib, a second-line tyrosine kinase inhibitor, however, 1 year later, developed further peritoneal and hepatic metastases.

### Case 8

A 52-year-old male presented with rectal bleeding. A CT detected a rectal mass and liver lesions. He underwent an anterior resection and was found to have a rectal GIST. He was commenced on imatinib which resulted in a decrease in attenuation and size of the liver metastases. 20 months after commencing tyrosine kinase inhibitors, surveillance CT imaging demonstrates soft tissue nodularity in one of the cystic attenuating metastases. This was not reported at the time and a subsequent CT 3 months later shows further increase in size of the soft tissue nodule and new enhancement in a further lesion indicating recurrent hepatic metastatic disease (Figure 8).

## Conclusion

A wide range of appearances of GIST liver metastases prior to, during and after treatment may be seen on CT and MRI. GIST liver metastases may be hypo- or hypervascular in the arterial phase and occasionally isoenhancing, relative to the background liver parenchyma, in the portal venous phase. As a result, they may potentially be missed if portal venous imaging alone is performed. After treatment with tyrosine kinase inhibitors, liver metastases often appear cystic, frequently without size reduction. Recurrent liver metastases may be heralded by increased vascularity or the development of an internal solid component within a cystic lesion. This may occur without a discernible increase in lesion size. An awareness of these findings is essential in timely diagnosis, assessment of treatment response and detection of liver metastasis recurrence. This is of particular importance given the increasing use of targeted ablative treatment and surgical resection of GIST liver metastases.

## Learning points

- GIST genetic mutations predict response to molecular-targeted therapy and influence prognosis.
- Multiphase contrast imaging of the liver is required to detect GIST liver metastases, as arterial enhancing metastases may be isodense in the portal venous phase, and therefore difficult to detect if single-phase portal venous imaging is performed.
- Surveillance multiphase contrast CT imaging is useful to monitor changes in tumour vascularity before and after treatment.
- Tumour characteristics are more indicative of tumour response rather than size. After treatment with tyrosine kinase inhibitors, liver metastases often appear cystic, frequently without shrinkage. Pseudoprogression may be seen post-treatment with an increase in lesion size.
- Recurrent liver metastases may be heralded by increased vascularity or the development of an internal solid component within a cystic lesion which may occur without a discernible increase in lesion size.
